# Comparison of Seegene Anyplex II HPV28 assay with BD Onclarity HPV assay for human papillomavirus genotyping

**DOI:** 10.1371/journal.pone.0267836

**Published:** 2022-07-08

**Authors:** Moonsik Kim, Jinhee Kim, Nora Jee-Young Park, Ji Young Park

**Affiliations:** Department of Pathology, School of Medicine, Kyungpook National University, Kyungpook National University Chilgok Hospital, Daegu, Republic of Korea; University of California, San Francisco, UNITED STATES

## Abstract

Presently, human papillomavirus (HPV)-based cervical cancer screening is commonly used and is replacing conventional cytology screening tests. The HPV genotyping assay is useful for triage in cervical cancer screening and the evaluation of HPV vaccination effects. In this study, we evaluated the clinical performance of two HPV genotyping assays, BD Onclarity HPV (Onclarity) and Seegene Anyplex II HPV28 (Anyplex) in the detection of relevant cervical lesions and for HPV genotyping concordance. Anyplex and Onclarity assays were performed on 920 consecutive liquid-based specimens. Anyplex, sensitivity, specificity, and genotyping concordance with Onclarity were optimal when restricted to ≥2+ (medium) viral loads. HPV genotyping agreement between the two assays ranged between 0.75 and 0.9 (excellent), except for HPV 33/58, which was 0.73 (good). With Onclarity as a reference, the relative sensitivity of Anyplex for the detection of ≥CIN 2 was 1.05 (95% CI: 0.99–1.1) and the relative specificity for detection of negative for intraepithelial lesion and malignancy (NILM) was 0.89 (95% CI: 0.85–0.93). For most ≥CIN 2 lesions, high-risk HPV was detected by Onclarity (66/72) and Anyplex (69/72) assays. For high-risk HPV negative ≥CIN 2 lesions, possible high-risk HPV genotypes were detected by Anyplex. In conclusion, the genotyping agreement between the tests was good to excellent. Full genotyping with Anyplex might confer additional benefits to patients with ≥CIN 2, although the difference is small. We also suggest an optimal cutoff value when reporting HPV infections using the Anyplex assay (≥2+; medium viral loads).

## Introduction

The majority of cervical cancers and their precursor lesions are derived from human papillomavirus (HPV) infections [1]. Thus far, over 200 HPV genotypes have been identified [2] and 14 anogenital HPVs have been categorized as high-risk HPV (hrHPV) types (HPV 16, 18, 31, 33, 35, 39, 45, 51, 52, 56, 58, 59, 66, and 68) [3]. HPV 26, 53, 69, 73, and 82 have also been associated with cervical cancer in previous studies [3, 4]. HPV vaccines are widely used to prevent cervical cancer and greatly reduce cervical cancer and its precursor lesions. Currently, bivalent (HPV 16 and HPV 18), quadrivalent (HPV 16, HPV 18, HPV 6, and HPV 11), and 9-valent (HPV 16, HPV 18, HPV 6, HPV 11, HPV 31, HPV 33, HPV 45, HPV 52, and HPV 58) vaccines are commercially available [5, 6]. Thus, understanding the genotypes and distribution of HPV is essential to establish preventative programs, including vaccination.

HPV-based cervical cancer screening is widely used and has superior sensitivity and negative predictive value compared with cytology screening tests [7]. First-generation HPV assays (the Amplicor HPV test and Linear Array HPV genotyping test) were able to provide test results as either HPV positive or negative, without information about the specific HPV genotype [8, 9]. Over the last decade, HPV assays have undergone a rapid evolution and newer generations of HPV assays can detect individual HPV genotypes. The current commercially available HPV assays can be classified into three categories: 1) limited genotyping, including the identification of HPV 16 and HPV 18, with pooled detection of the remaining oncogenic HPV genotypes; 2) extended HPV genotyping, which detects ≥5 genotypes (including HPV 16 and HPV 18) combined with one or more bulk detections of the remaining oncogenic HPV genotypes; and 3) full genotyping assays, which identify all individual oncogenic HPVs [10].

The BD Onclarity^TM^ HPV assay (Onclarity) is an FDA-approved HPV test that uses real-time polymerase chain reaction (PCR) technology. It targets specific E6 and E7 gene regions. Onclarity can detect six individual genotypes (HPV 16, 18, 31, 45, 51, and 52) and three distinct groups comprising a total of eight HPV genotypes (HPV 33 and 58; HPV 56, 59, and 66; and HPV 35, 39, and 68) [11]. The Seegene Anyplex II HPV28 assay (Anyplex) is a semiquantitative, multiplex, real-time PCR assay. It has been specifically designed to simultaneously detect 14 hrHPV types (HPV 16, 18, 31, 33, 35, 39, 45, 51, 52, 56, 58, 59, 66, and 68), five possible hrHPV types (HPV26, 53, 69, 73, and 82), and nine low-risk (lr)HPV types (HPV 6, 11, 40, 42, 43, 44, 53, 54, and 70) [12]. Although clinical validation of Onclarity and Anyplex has been performed in previous studies according to the international guidelines for human papillomavirus DNA testing [13–17], a direct comparison of these two tests focused on genotyping capabilities has not yet been conducted.

In this study, we investigated the abilities of the two assays to detect clinically relevant cervical lesions and compared their relative sensitivity and specificity. We also analyzed the HPV genotype-specific concordance between the assays. Optimal cutoff value when reporting HPV infections using the Anyplex assay was also evaluated.

## Materials and methods

### Study population

We retrospectively gathered 920 consecutive liquid-based specimens taken at the Department of Pathology, Kyungpook National University Chilgok Hospital, Daegu, between 2016 and 2019. Patients visited the hospital for health check-ups, biopsy results, and because of abnormal cytology test results. Demographic and clinicopathological data including age, cytology results, and biopsy results were retrieved from the medical records. The Institutional Review Board approved the study (KNUCH 2019-04-002-002). Requirement of written informed consent from the patients was waived due to the retrospective nature of this research.

### Cytological testing

Liquid-based Papanicolaou tests (SurePath; BD, Franklin Lakes, N.J., USA) were performed for cytological evaluation. Cytological findings were interpreted following the 2001 Bethesda System for cervicovaginal cytology reporting [18]. All cytology screenings were performed using Papanicolaou tests. The results of these tests were interpreted by two experienced gynecological pathologists (JYP and NJP) at Kyungpook National University Chilgok Hospital.

### HPV genotyping

HPV genotyping with Anyplex was performed using the Anyplex II HPV 28 assay kit (Seegene, Korea) with cervicovaginal swab specimens. DNA extraction and then the Anyplex assays were carried out following the manufacturer’s instructions. Briefly, 5 μL of DNA was used in each of the two 20-μL reactions with primer set A or B. In the assay, HPV-specific dual priming oligonucleotides were used for multiplex (real-time) PCR. A total of 28 HPV types were tested to simultaneously detect 14 hrHPV types (HPV 16, 18, 31, 33, 35, 39, 45, 51, 52, 56, 59, 66, and 68), five possible hrHPV types (HPV26, 58, 69, 73, and 82), and nine lrHPV types (HPV 6, 11, 40, 42, 43, 44, 53, 54, and 70). Viral loads were semiquantified in Anyplex as 1+ (low viral load—positive signal at ≥40 PCR cycles), 2+ (medium viral load—positive signal between 31 and 39 PCR cycles), or 3+ (high viral load—positive signal <31 PCR cycles).

Onclarity testing uses the automated Viper Lt platform, the full workflow for which has been described previously in detail [19]. Briefly, 0.5 ml of original resuspended SurePath material was aliquoted to a BD tube containing 1.7 ml of sample medium. The samples were prewarmed for 30 min at 120°C. Then, they were transferred to the fully automated Viper Lt platform and tested with Onclarity according to the manufacturer’s instructions.

### Pathological evaluation

Of the 920 cases subjected to HPV genotyping, 203 cases underwent colposcopy-guided cervical biopsy according to the 2012 American Society for Colposcopy and Cervical Pathology guidelines [20]; first, cervical cytology results corresponding to atypical squamous cells of undetermined significance (ASCUS) or worse, and second, HPV16/18 positivity with negative cervical cytology results.

Pathological diagnoses of the tissue specimens were independently reviewed by two gynecological pathologists—JYP and NJP—in a blinded manner. Cases with discrepant results were repeatedly reviewed until a consensus was reached.

### Statistical analysis

Relationships between clinicopathologic parameters were evaluated using the Chi-square test for categorical parameters and Fisher’s exact test for those with an expected frequency of less than 5. A p value of<0.05 was considered to indicate a significant difference. Tables for relative sensitivity and specificity were constructed using the assay results. The levels of genotyping agreement between Anyplex and Onclarity were determined using kappa statistics. A kappa range below 0.40 was interpreted as poor agreement, 0.40–0.59 was fair, 0.60–0.74 was good, and 0.75–1.00 was excellent. Statistical differences were considered significant at p < 0.05. All statistical analyses were conducted using IBM SPSS v.23 (IBM, Armonk, NY, USA).

## Results

### Clinicopathological and HPV characteristics of the patient cohort

The mean age of patients in the sample was 55.4 years (range: 30–87). The cytological categorization of the patient group was as follows: 592 were negative for intraepithelial lesions or malignancies (NILM); 160 had ASCUS, 72 had low-grade squamous intraepithelial lesions (LSIL); 26 had high-grade squamous intraepithelial lesions (HSIL)+ (HSIL or squamous cell carcinoma); and 14 had either atypical glandular cells (AGC), atypical squamous cells for which HSIL could not be excluded (ASC-H), or adenocarcinoma *in situ* (AIS). Of the 203 cases that underwent colposcopy-guided cervical biopsies, 69 were NILM, 62 had cervical interepithelial neoplasia (CIN) 1, 13 had CIN 2, 37 had CIN 3, and 22 had cervical cancer. There were 72 cases of ≥CIN 2. The Onclarity assays showed hrHPV positivity in 49.8% (458/920) of the patient group. When restricted to a ≥2+ (medium) viral load, 509 of the 920 cases (55.3%) were hrHPV positive in the Anyplex assays. HrHPV was detected more frequently by Anyplex in the overall population. However, there was no significant difference between the two assays for the detection of HSIL+ (cytology) and ≥CIN2 (histology), which have clinical importance. The hrHPV detection rate was highest for women aged ≥60 years with both Onclarity (59.8%) and Anyplex (64.7%). More detailed information on the patient cohort is shown in Table 1.

**Table 1:** Characteristics of the study population and prevalence of hrHPV assessed by Onclarity and Anyplex (≥2+ viral load).

	Total	Onclarity assay	Anyplex	p-value
		hr-HPV pos	hr-HPV pos	
All	920	458 (49.8%)	509 (55.3%)	0.020
Age				
30–39	98	42 (42.9%)	53 (54.1%)	0.153
40–49	184	75 (40.8%)	83 (45.1%)	0.461
50–59	315	148 (46.9%)	164 (52.1%)	0.232
≥60	323	193 (59.8%)	209 (64.7%)	0.223
Cytology				
N/A	56	46 (82.1%)	47 (83.9%)	1.000
NILM	592	224 (37.8%)	264 (44.5%)	0.021
ASCUS	160	106 (66.2%)	109 (68.1%)	0.721
LSIL	72	49 (68.1%)	53 (73.6%)	0.463
HSIL+	26	23 (88.5%)	26 (100.0%)	0.235
AGC/ASC-H/AIS	14	10 (71.4%)	10 (71.4%)	1.000
Histological Diagnosis				
N/A	717	321 (44.8%)	361 (50.3%)	0.039
NILM	69	30 (43.5%)	33 (47.8%)	0.733
CIN1	62	41 (66.1%)	46 (74.2%)	0.433
CIN2	13	13 (100.0%)	13 (100.0%)	1.000
CIN3	37	32 (86.5%)	35 (94.6%)	0.430
Cancer	22	21 (95.5%)	21 (95.5%)	1.000
≥CIN2	72	66 (91.7%)	69 (95.8%)	0.494

AGC: atypical glandular cells, AIS: adenocarcinoma in situ, ASC-H: atypical squamous cells, cannot rule out high-grade squamous intraepithelial lesion, ASCUS: atypical squamous cells of undetermined significance, CIN: cervical intraepithelial neoplasia, hr: high-risk, HSIL: high grade squamous intraepithelial lesion, LSIL: low grade squamous intraepithelial lesion, N/A: not available, NILM: negative for intraepithelial lesion or malignancy, pos: positive

**Fig 1:**
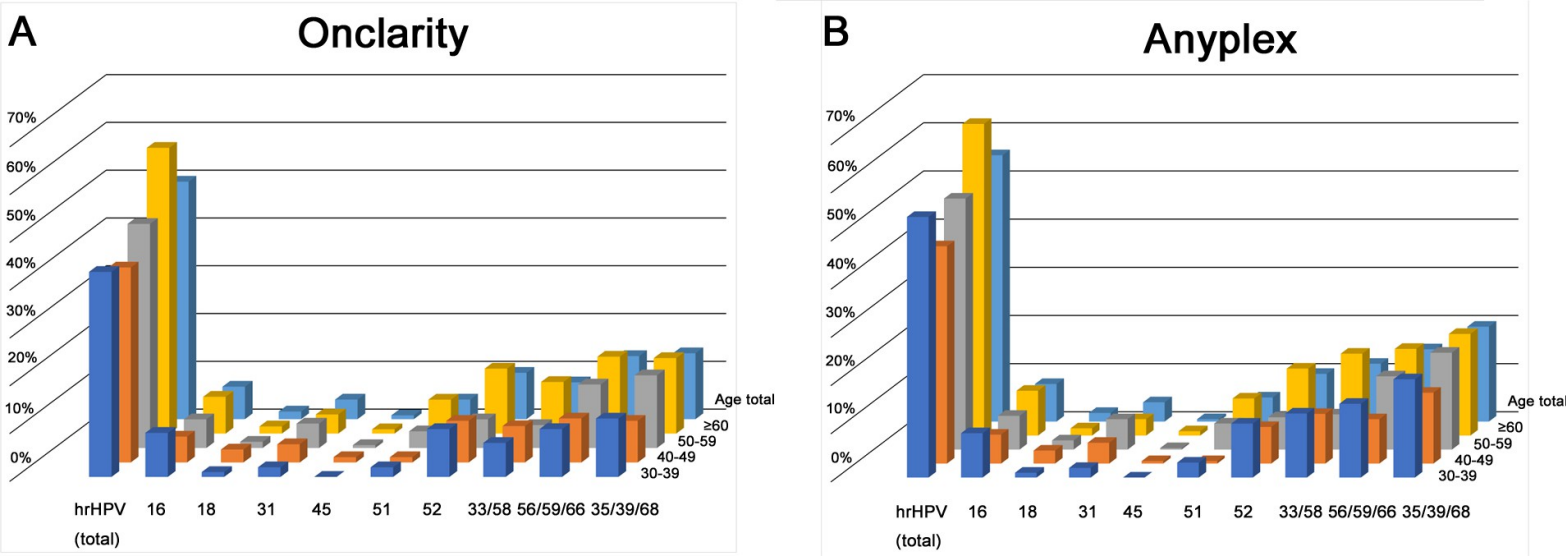
HPV genotyping prevalence comparison by age group. (A) Onclarity and (B) Anyplex.

The prevalence of HPV 16, 18, 31, 45, 51, and 52 were 6.8%, 1.6%, 4.1%, 0.8%, 4.1%, and 9.7%, respectively, in Onclarity and 7.8%, 1.8%, 4.0%, 0.5%, 5.0%, and 9.9%, respectively, in Anyplex. The prevalence of HPV 33/58, 56/59/66, and 35/39/68 were 7.7%, 13.2%, and 13.8%, respectively, in Onclarity. When the individual HPV genotypes from Anyplex assays were categorized into three groups for comparison with the bulk groups in the Onclarity assays, Anyplex detected 12.0% (HPV 33/58), 15.0% (56/59/66), and 19.7% (35/39/68) hrHPV (Fig 1 and Table 2).

**Table 2:** HPV genotyping prevalence by age assessed by Onclarity and Anyplex (≥2+ viral load).

	Age				
Assay and HPV Type	30–39 (98)	40–49 (184)	50–59 (315)	≥60 (323)	Total (920)
	Onclarity				
hrHPV	42 (42.9%)	75 (40.8%)	148 (46.9%)	193 (59.8%)	458 (49.7%)
16	9 (9.2%)	10 (5.4%)	19 (6.0%)	25 (7.7%)	63 (6.8%)
18	1 (1.0%)	5 (2.7%)	4 (1.3%)	5 (1.5%)	15 (1.6%)
31	2 (2.0%)	7 (3.8%)	16 (5.1%)	13 (4.0%)	38 (4.1%)
45	0 (0.0%)	2 (1.1%)	2 (0.6%)	3 (0.9%)	7 (0.8%)
51	2 (2.0%)	2 (1.1%)	11 (3.5%)	23 (7.1%)	38 (4.1%)
52	10 (10.0%)	16 (8.7%)	19 (6.0%)	44 (13.6%)	89 (9.7%)
33/58	7 (7.1%)	14 (7.6%)	15 (4.8%)	35 (10.8%)	71 (7.7%)
56/59/66	10 (10.0%)	17 (9.2%)	42 (13.3%)	52 (16.1%)	121 (13.2%)
35/39/68	12 (12.2%)	16 (8.7%)	48 (15.2%)	51 (15.8%)	127 (13.8%)
	Anyplex				
hrHPV	53 (54.1%)	83 (45.1%)	164 (52.1%)	209 (64.7%)	509 (55.3%)
16	9 (9.2%)	11 (6.0%)	22 (7.0%)	30 (9.3%)	72 (7.8%)
18	1 (1.0%)	5 (2.7%)	6 (1.9%)	5 (1.5%)	17 (1.8%)
31	2 (2.0%)	8 (4.3%)	16 (6.3%)	11 (3.4%)	37 (4.0%)
45	0 (0.0%)	1 (0.5%)	1 (0.3%)	3 (0.9%)	5 (0.5%)
51	3 (3.1%)	1 (0.5%)	17 (5.4%)	25 (7.7%)	46 (5.0%)
52	11 (11.2%)	14 (7.6%)	21 (6.7%)	45 (13.9%)	91 (9.9%)
33/58	13 (13.3%)	19 (10.3%)	23 (7.3%)	55 (17.0%)	110 (12.0%)
56/59/66	15 (15.3%)	17 (9.2%)	48 (15.2%)	58 (18.0%)	138 (15.0%)
35/39/68	20 (20.4%)	27 (14.7%)	66 (20.1%)	68 (21.1%)	181 (19.7%)

**Fig 2:**
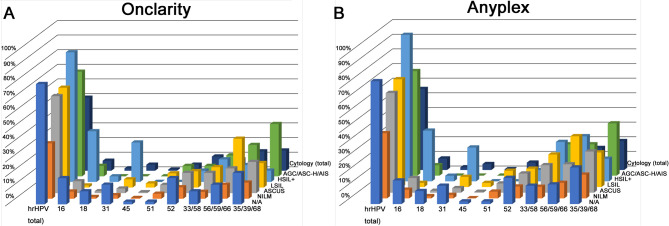
HPV genotyping prevalence comparison by cytology. (A) Onclarity and (B) Anyplex.

In the cytology samples, the hrHPV detection rate by Onclarity was highest for HSIL+, AGC/ASC-H/AIS, LSIL, ASC-US, and NILM, in descending order. In Anyplex, hrHPV was identified most frequently in HSIL+, LSIL, AGC/ASC-H/AIS, ASC-US, and NILM, in descending order (Fig 2 and Table 3). The Anyplex HPV genotyping results of ≥1+ (low viral load) or ≥3+ (high viral load) are shown in the S1–S6 Tables.

**Table 3:** HPV genotyping prevalence for Onclarity and Anyplex (≥2+ viral load) stratified by cytology results.

	Cytology						
Assay and HPV Type	N/A (56)	NILM (592)	ASC-US (160)	LSIL (72)	HSIL+ (26)	AGC/ASC-H/AIS (14)	Total (920)
Onclarity							
hrHPV	46 (82.1%)	224 (37.8%)	106 (66.2%)	49 (68.1%)	23 (88.5%)	10 (71.4%)	458 (49.8%)
16	10 (17.9%)	29 (4.9%)	13 (8.1%)	1 (1.4%)	9 (34.6%)	1 (7.1%)	63 (6.8%)
18	5 (8.9%)	7 (1.2%)	2 (1.3%)	0 (0.0%)	1 (3.8%)	0 (0.0%)	15 (1.6%)
31	8 (13.4%)	14 (2.4%)	5 (3.1%)	4 (5.6%)	7 (26.9%)	0 (0.0%)	38 (4.1%)
45	1 (1.8%)	3 (0.5%)	1 (0.6%)	2 (2.8%)	0 (0.0%)	0 (0.0%)	7 (0.8%)
51	1 (1.8%)	20 (3.4%)	9 (5.6%)	7 (9.7%)	0 (0.0%)	1 (7.1%)	38 (4.1%)
52	10 (17.9%)	46 (7.8%)	22 (13.8%)	8 (11.1%)	2 (7.7%)	1 (7.1%)	89 (9.7%)
33/58	5 (8.9%)	28 (4.7%)	22 (13.8%)	10 (13.9%)	4 (15.4%)	2 (14.3%)	71 (7.7%)
56/59/66	8 (13.4%)	56 (9.5%)	27 (16.9%)	24 (33.3%)	3 (11.5%)	3 (21.4%)	121 (13.2%)
35/39/68	12 (21.4%)	62 (11.0%)	34 (21.3%)	12 (16.7%)	2 (7.7%)	5 (35.7%)	127 (13.8%)
Anyplex							
hrHPV	47 (83.9%)	264 (44.5%)	109 (68.1%)	53 (73.6%)	26 (100.0%)	10 (71.4%)	509 (55.3%)
16	9 (16.1%)	35 (5.9%)	16 (10.0%)	2 (2.8%)	9 (34.6%)	1 (7.1%)	72 (7.8%)
18	5 (8.9%)	9 (1.5%)	2 (1.3%)	0 (0.0%)	1 (3.8%)	0 (0.0%)	17 (1.8%)
31	7 (12.5%)	14 (2.4%)	5 (3.1%)	5 (6.9%)	6 (23.1%)	0 (0.0%)	37 (4.0%)
45	1 (1.8%)	1 (0.1%)	1 (0.6%)	2 (2.8%)	0 (0.0%)	0 (0.0%)	5 (0.5%)
51	1 (1.8%)	27 (4.6%)	10 (6.3%)	8 (11.1%)	0 (0.0%)	0 (0.0%)	46 (5.0%)
52	10 (17.9%)	48 (8.1%)	21 (13.1%)	9 (12.5%)	2 (7.7%)	1 (7.1%)	91 (9.9%)
33/58	7 (12.5%)	47 (7.9%)	30 (18.8%)	16 (22.2%)	7 (26.9%)	3 (21.4%)	110 (12.0%)
56/59/66	8 (13.4%)	63 (10.6%)	31 (19.4%)	25 (34.7%)	8 (30.8%)	3 (21.4%)	138 (15.0%)
35/39/68	14 (25.0%)	96 (16.2%)	45 (28.1%)	17 (23.6%)	4 (15.4%)	5 (35.7%)	181 (19.7%)

### Clinical performance of Anyplex and Onclarity

Then, we compared the clinical performance of Anyplex and Onclarity (Table 4). The sensitivity and specificity of Anyplex compared with that of Onclarity was optimal when restricted to ≥2+ (medium) viral loads. In the Anyplex assay, 69 of the 72 ≥CIN 2 cases were reported to be hrHPV positive when restricted to a ≥2+ (medium) viral load. The Onclarity assay reported 66 of the 72 ≥CIN 2 cases to be hrHPV positive. The relative sensitivity of Anyplex was 1.05 (95% confidence interval [CI]: 0.99–1.1) of that of Onclarity. The relative sensitivity of Onclarity was 0.96 (95% CI: 0.91–1.01) of that of Anyplex. From a total of 592 NILM cases, Onclarity determined 368 cases to be hrHPV negative, whereas Anyplex determined 328 cases to be hrHPV negative. The relative specificity of Anyplex was 0.89 (95% CI: 0.85–0.93) of that of Onclarity. The relative specificity of Onclarity for the detection of NILM was 1.12 (95% CI: 1.07–1.17) of that of Anyplex. The relative sensitivity and specificity of Anyplex compared with that of Onclarity for ≥1+ (low viral load) and ≥3+ (high viral load) are shown in the S7 and S8 Tables.

**Table 4:** Clinical accuracy of Onclarity and Anyplex (≥2+ viral load) for ≥CIN2, ≥CIN3, and NFM outcomes.

		Anyplex Results			Onclarity	Anyplex
Study Population	Onclarity Results	Pos.	Neg.	Total		
≥CIN2 (N = 72)	Pos.	66	0	66	Sensitivity 95.6% (CI: 87.8–99.1)	Sensitivity 100.0% (CI: 94.6–100.0)
	Neg.	3	3	6		
	Total	69	3	72		
	Relative Sensitivity ≥CIN2 1.05 (0.99–1.1) (Onclarity as a reference)					
≥CIN3 (N = 59)	Pos.	53	0	53	Sensitivity 94.6% (CI: 85.1–98.9)	Sensitivity 100.0% (CI: 93.3–100.0)
	Neg.	3	3	6		
	Total	56	3	59		
	Relative Sensitivity ≥CIN3 1.06 (0.99–1.12) (Onclarity as a reference)					
NFM (N = 592)	Pos.	212	12	224	Specificity 96.3% (CI: 93.7–98.1)	Specificity 85.9% (CI: 81.9–89.3)
	Neg.	52	316	368		
	Total	264	328	592		
	Relative Specificity NFM 0.89 (0.85–0.93) (Onclarity as a reference)					

### HPV genotyping concordance between the Anyplex and Onclarity

HPV genotyping concordance was best when Anyplex was restricted to a ≥2+ (medium) viral load. Kappa values were good to excellent (range: 0.73–0.9) for concordance between the Anyplex and Onclarity assays. The kappa values were excellent for HPV 16, 18, 31, 45, 51, 52, 35/39/68, and 56/59/66 and good for HPV 33/58 (kappa value: 0.73). The agreement for the 14 hrHPV combined was 90.1% (kappa value: 0.8) (Table 5). The HPV genotyping concordances for ≥1+ (low viral load) and ≥3+ (high viral load) between Anyplex and Onclarity are shown in the S9 and S10 Tables.

**Table 5:** Detection of individual oncogenic genotypes by Onclarity and Anyplex (≥2+ viral load).

HPV Genotypes	Population (n = 920)							
	Onc+	Anyplex+	Onc+/Anyplex+	Onc+/Anyplex−	Onc−/Anyplex+	Onc−/Anyplex−	Agreement	Kappa
16	63 (6.8%)	72 (7.8%)	61	2	11	846	98.6	0.9
18	15 (1.6%)	17 (1.8%)	14	1	3	902	99.6	0.87
31	38 (4.1%)	37 (4.0%)	34	4	3	879	99.2	0.9
45	7 (0.8%)	5 (0.5%)	5	2	0	913	99.8	0.83
51	38 (4.1%)	46 (5.0%)	35	3	11	871	98.5	0.83
52	89 (9.7%)	91 (9.9%)	79	10	12	819	97.6	0.86
33/58	71 (7.7%)	110 (12.0%)	68	3	42	807	95.1	0.73
56/59/66	121 (13.2%)	138 (15.0%)	111	10	27	772	96	0.83
35/39/68	127 (13.8%)	181 (19.7%)	121	6	60	733	93.8	0.75
14 hrHPV	458 (49.8%)	509 (55.3%)	437	19	72	390	90.1	0.8

## Discussion

In this study, we compared the clinical and type-specific performance of Anyplex and Onclarity assays. Both tests demonstrated excellent HPV DNA genotyping concordance, except for HPV 33/58. Anyplex showed similar clinical sensitivity (relative sensitivity: 1.05, 95% CI: 0.99–1.1) for the detection of ≥CIN 2 lesions to Onclarity. The specificity of Anyplex was somewhat lower than that of Onclarity for the detection of NILM. Agreement between the two assays was highest when reporting HPV infection cases with ≥2+ (medium) viral loads, rather than ≥1+ (low) viral loads or ≥3+ (high) viral loads. To the best of our knowledge, this is the first study that directly compares the clinical performance and genotyping capabilities of Anyplex and Onclarity.

Currently, “Guidelines for human papillomavirus DNA test requirements for primary cervical cancer screening in women of 30 years and older” [21] are widely accepted and used to validate HPV assays. However, these guidelines primarily focus on the overall ability of HPV assays to detect clinically relevant hrHPV infections. Measuring HPV infections only at the level of “positive or negative” cannot provide information about the individual HPV genotype. Although HPV16 and HPV18 constitute >70% of cervical cancers [3, 22], previous studies suggest that a wider range of HPV genotyping may confer additional clinical benefits [23, 24]. HPV genotyping is also needed to triage patients for cervical cancer screening [25], distinguish transient HPV infections from permanent HPV infections [26], and establish the impact of HPV vaccination programs [27, 28].

Onclarity is an FDA-approved HPV genotyping test widely used for cervical cancer screening including several major hospitals at South Korea [29]. It can detect 14 hrHPV genotypes (HPV 16, 18, 31, 45, 51, 52, 33/ 58, 56/59/66, and 35/39/68). Anyplex offers complete genotyping for 28 HPVs including hrHPVs, possible hrHPVs, and lrHPVs. Anyplex is also widely used for cervical cancer screening in South Korea, including at our hospital [30–32]. Meanwhile, patients tend to visit different hospitals and may repetitively undergo similar HPV genotyping assays due to relatively lower costs of national health insurance in Korea [33–35]. Therefore, a comparison of the clinical performance of these widely used tests may provide consistent information with regard to HPV infection status and avoid redundant genotyping tests. Therefore, we validated the comprehensive performance of these two HPV assays, focusing on HPV genotyping capability and the accurate detection of clinically relevant ≥CIN2 lesions.

We found excellent concordance between the assays in HPV genotyping except for the genotyping of HPV 33/58, for which there was good concordance. Some of the discrepant results could be attributed to differences in the assay technologies. Although Onclarity targets the E6/E7 gene of HPV [14], Anyplex targets the L1 gene [36]. Moreover, Onclarity has an amplification target range from 79 to 137 base pairs, whereas Anyplex has a target range of ~150 base pairs [36]. Furthermore, agreement in HPV genotyping was lower for HPV 33/58, 56/59/66, and 35/39/68. Although Onclarity detects these genotypes in three bulk groups, Anyplex identifies all HPV genotypes individually. Altogether, these differences in detection technologies might affect the concordance between the assays.

Although hrHPV was detected for the majority of ≥CIN 2 lesions by both assays, there were a few ≥CIN 2 cases for which hrHPV was not detected. Using the Onclarity assay, six ≥CIN 2 cases were negative for hrHPV (6/72). Using Anyplex, three cases were negative for hrHPV (3/72) (HPV infections ≥2+ (medium) viral load). For these three cases, however, HPV 53 and 69—possible hrHPV groups that were not included in the Onclarity panel—and HPV 16 infection with a 1+ (low) viral load were detected by Anyplex upon subsequent analysis. Thus, Anyplex may confer additional benefits in the management of patients with ≥CIN 2 lesion, although the difference with Onclarity is small.

The specificity of Anyplex was relatively low in comparison to the Onclarity assay. However, this can be partially explained by the characteristics of the patient cohort. As our sample was drawn from the patient records of a tertiary hospital, the majority of the patients in our study were referred to the hospital due to abnormal cytology, biopsy, or HPV genotyping results obtained at a local clinic rather than for routine screening purposes. Thus, the high prevalence of HPV infection in the study cohort might have affected the specificity of the Anyplex assays.

Anyplex is a semiquantitative real-time PCR assay that categorizes HPV viral load as 1+ (low), 2+ (medium), or 3+ (high). However, there is no consensus as to the cutoff point for optimal sensitivity, specificity, and genotyping accuracy in the Anyplex assay. In this study, reporting all HPV infections with 1+ to 3+ viral load yielded only a small increase in detection sensitivity for ≥CIN 2 lesions, whereas the specificity decreased significantly. When the reported HPV infections were restricted to those with a 3+ viral load, there was a significant decrease in detection sensitivity for ≥CIN 2 lesions. The genotyping concordance between Anyplex and Onclarity was also highest when reporting was restricted to ≥2+ viral loads with Anyplex. Therefore, it is suggested that reporting ≥2+ viral load is optimal for cervical cancer screening using Anyplex.

This study had some limitations. First, we did not perform the additional reference genotyping assay such as HC2 or GP5+/6+ PCR to estimate the accuracy of the HPV genotyping tests. However, previous studies of these two assays using the non-inferiority test have validated their clinical performance for cervical cancer screening [13, 37]. Subsequent studies might be needed to assess the discordant results between the two assays presented in this study. Furthermore, the study sample was from a tertiary referral hospital, many patients visited the hospital due to the abnormal cytology, biopsy, or HPV genotyping test results. This might have influenced the specificity of the Anyplex and Onclarity assays in this study. A larger cohort study from a cervical cancer screening program that also incorporates a control population is warranted to confirm the specificity of the two assays. As Onclarity only provides information on the hrHPVs, comparisons with other full HPV genotyping assays should be performed in subsequent studies to validate the genotyping accuracy of Anyplex.

In conclusion, we demonstrated that Onclarity and Anyplex had excellent genotyping concordance in general. Although both assays were able to detect most clinically relevant ≥CIN 2 lesions, extensive genotyping by Anyplex may benefit additional patients with ≥CIN 2 lesions. Reporting ≥2+ viral load in Anyplex was optimal for sensitivity, specificity, and genotyping concordance in this study. Subsequent studies should be performed to verify our findings.

## Supporting information

S1 Table Characteristics of the study population and prevalence of high-risk HPV assessed by Onclarity and Anyplex (1+ low viral load). (XLSX)

S2 Table Characteristics of the study population and prevalence of high-risk HPV assessed by Onclarity and Anyplex (3+ high viral load). (XLSX)

S3 Table HPV genotyping prevalence by age assessed by Onclarity and Anyplex (1+ low viral load). (XLSX)

S4 Table HPV genotyping prevalence by age assessed by Onclarity and Anyplex (3+ high viral load). (XLSX)

S5 Table HPV genotyping prevalence with Onclarity and Anyplex (1+ low viral load) stratified by cytology result. (XLSX)

S6 Table HPV genotyping prevalence for Onclarity and Anyplex (3+ high viral load) stratified by cytology results. (XLSX)

S7 Table Clinical accuracy of Onclarity and Anyplex (1+ low viral load) for ≥CIN2, ≥CIN3, and NFM outcomes. (XLSX)

S8 Table Clinical accuracy of Onclarity and Anyplex (3+ high viral load) for ≥CIN2, ≥CIN3, and NFM outcomes. (XLSX)

S9 Table Detection of individual oncogenic genotypes by Onclarity and Anyplex (1+ low viral load). (XLSX)

S10 Table Detection of individual oncogenic genotypes by Onclarity and Anyplex (3+ high viral load). (XLSX)
